# The DELLA Proteins Influence the Expression of Cytokinin Biosynthesis and Response Genes During Nodulation

**DOI:** 10.3389/fpls.2019.00432

**Published:** 2019-04-09

**Authors:** Alexandra V. Dolgikh, Anna N. Kirienko, Igor A. Tikhonovich, Eloise Foo, Elena A. Dolgikh

**Affiliations:** ^1^All-Russia Research Institute for Agricultural Microbiology, St. Petersburg, Russia; ^2^School of Natural Sciences, University of Tasmania, Hobart, TAS, Australia

**Keywords:** legume-rhizobium symbiosis, root nodule development, mutants, cytokinins, gibberelllins, crosstalk

## Abstract

The key event that initiates nodule organogenesis is the perception of bacterial signal molecules, the Nod factors, triggering a complex of responses in epidermal and cortical cells of the root. The Nod factor signaling pathway interacts with plant hormones, including cytokinins and gibberellins. Activation of cytokinin signaling through the homeodomain-containing transcription factors KNOX is essential for nodule formation. The main regulators of gibberellin signaling, the DELLA proteins are also involved in regulation of nodule formation. However, the interaction between the cytokinin and gibberellin signaling pathways is not fully understood. Here, we show in *Pisum sativum* L. that the DELLA proteins can activate the expression of KNOX and BELL transcription factors involved in regulation of cytokinin metabolic and response genes. Consistently, pea *la cry-s* (*della1 della2*) mutant showed reduced ability to upregulate expression of some cytokinin metabolic genes during nodulation. Our results suggest that DELLA proteins may regulate cytokinin metabolism upon nodulation.

## Introduction

Most legume plants are able to form symbiosis with nitrogen-fixing bacteria that results in a new organ formation, the nodule. Multiple studies have shown that root nodule formation is regulated by several plant hormones (reviewed by Ferguson and Mathesius, 2014; Gamas et al., 2017). However, the mechanisms of this regulatory network remain poorly understood. The essential role of cytokinins (CKs) in symbiosis development and their interplay with Nod factor-activated symbiotic signaling pathway has been shown. Indeed, in loss-of-function *Ljlhk1* and *Mtcre1* mutants defective in the receptors to CKs, a significant decrease in the number of nodules was found (Gonzalez-Rizzo et al., 2006; Murray et al., 2006; Plet et al., 2011). It was also shown that gain-of-function mutants in genes encoding receptors to CKs have an ability to form spontaneous nodule structures in the absence of compatible bacteria and exogenous application of CKs mimics the effect of Nod factor treatment in root cortex (Cooper and Long, 1994; Tirichine et al., 2007; Heckmann et al., 2011; Ovchinnikova et al., 2011). At the same time it has been found that CKs can play both positive and negative role in symbiosis depending on place, time and surrounding phytohormonal network (Gamas et al., 2017). This means that CK-activated signaling must be tightly regulated during nodulation and may interact with other phytohormonal signaling pathways during nodulation.

The gibberellins (GAs) are well-known phytohormones control many aspects of plant growth and development (Brian, 1959; Harberd et al., 1998). GAs have also been shown to play an important role during nodulation. Indeed, application and genetic studies indicate GAs can have both positive and negative effects of the number of nodules formed (e.g., Ferguson, 2005, Ferguson et al., 2011; Maekawa et al., 2009; Fonouni-Farde et al., 2016; Jin et al., 2016; McAdam et al., 2018). Like CKs, GAs appear to act at various stages of infection and nodule development (McAdam et al., 2018). Considering the functions of GAs and CKs it is possible these hormones interact to influence nodule initiation and development.

The active forms of GAs are perceived by the GID1 (GA insensitive dwarf1) receptor that in turn binds to and activates the degradation of DELLA proteins, via 26S proteasome (Alvey and Harberd, 2005; Achard et al., 2006, 2007; Navarro et al., 2008). The DELLAs are transcriptional regulators that repress GA responses and thus the DELLA may be considered as negative regulators of GA-signaling (Sun, 2010; Hedden and Thomas, 2012). As a consequence, the level of DELLA proteins can be changed depending on different external and internal factors (Davière and Achard, 2013; Park et al., 2013). Indeed, DELLA proteins may mediate crosstalk with other hormonal signaling pathways, coordinating the plants growth with responses to various stimuli (Floss et al., 2016; Fonouni-Farde et al., 2017).

It is known that DELLA proteins do not have DNA-binding domain and their regulatory ability is based on the interaction with different transcription factors, whose activity can be stimulated or inhibited in such interactions (Sun and Gubler, 2004; Fleet and Sun, 2005). Previous studies in legume plants have shown that DELLA proteins may interact with some transcriptional regulators in Nod factor-activated signaling pathway and modulate their function (Floss et al., 2016; Fonouni-Farde et al., 2016; Jin et al., 2016). Indeed, an important role for DELLA proteins in nodulation is supported by low infection and nodule number observed in *della* mutants or loss-of-function transgenics across legume species (Fonouni-Farde et al., 2016; Jin et al., 2016; McAdam et al., 2018). This may be mediated by physical interaction of the DELLA proteins with key components of the Nod factor signaling pathway including IPD3/CYCLOPS, NSP2 and NF-YA1 (Fonouni-Farde et al., 2016; Jin et al., 2016). However, it is important to note that a positive role for GA in nodule organogenesis has also been reported, as severley GA-deficient mutants of pea form many infections but few nodules that are poorly developed (McAdam et al., 2018). In contrast, the few nodules that form on *della* mutants of pea and Medicago appear to be normal size and at least in pea have been shown to have similar function to wild type (McAdam et al., 2018).

In order to find possible mechanisms of interplay between GA- and CK-signaling pathways, we have examined how the main regulators of the GA-signaling, the DELLA proteins, may influence KNOTTED1-LIKE HOMEOBOX (KNOX) and BEL1-LIKE HOMEODOMAIN (BELL) transcription factors involved in control of CKs and GAs levels in plants. Plant KNOX and BELL proteins are transcription factors that belong to the three-amino-acid-loop-extension (TALE) superfamily (Byrne et al., 2003; Smith and Hake, 2003; Bao et al., 2004; Sharma et al., 2014). These transcription factors interact as heterodimeric complexes to regulate transcription of target genes (Bellaoui et al., 2001; Chen et al., 2004; Hake et al., 2004; Lin et al., 2013). It is known that KNOX transcription factors may control the levels of CKs and GAs via direct regulating of CK biosynthesis *IPT* gene (Yanai et al., 2005) as well as GA metabolic genes, such as *GA 20-OXIDASE* (*GA20ox*) and *GA 2-OXIDASE* (*GA2ox*) in shoot apical meristem (Jasinski et al., 2005). Moreover, we have recently reported that KNOX3 transcription factor may promote the expression of some *IPT* and *LOG* genes during symbiosis development in legume plants *M. truncatula* and pea *Pisum sativum* L. (Azarakhsh et al., 2015). Recent study has also revealed that GA signaling mediated by *Mt*DELLA1 decreases the amount of the free base CK content in roots of *M. truncatula* (Fonouni-Farde et al., 2017). However, the interaction between CKs and GAs during nodulation is still not known and the mechanisms of DELLAs’ impact on CKs status remain poorly understood.

Here we used the pea *La cry-s* (*DELLA1 della2*) and *la cry-s (della1 della2*) mutants to examine the effects of DELLA proteins on KNOX and BELL transcription factors. We have found that the up-regulation of expression of some *PsKNOX* and *PsBELL* genes seen in wild type was reduced in double *la cry-s* (*della1 della2*) mutant during initial and later stages of nodulation. Pea *la cry-s* (*della1 della2*) mutants also showed reduced capacity to elevate expression of the CK metabolic and signaling genes during nodulation compared to wild type. Therefore, *Ps*DELLA proteins may be involved in regulation of the CK metabolism during nodulation. To test if this action of DELLA was via KNOX and BELL transcription factors, the Y2H studies were undertaken but did not reveal a strong direct interaction between *Ps*DELLA1 and *Ps*KNOX3 or *Ps*BELL1. Therefore, *Ps*DELLA1 may promote the expression *PsKNOX3* and *PsBELL1* and their target CK metabolic and signaling genes indirectly through other known transcription factors and we begin to examine the role of *Ps*IPD3/CYCLOPS.

## Materials and Methods

### Bacterial Strains and Inoculation

Inoculation of the pea plants was conducted with the *Rhizobium leguminosarum* biovar *viciae* wild type strain 3841. Bacterial liquid culture was grown in B^-^ medium (van Brussel et al., 1977), diluted upto the optical density at 600 nm (OD_600_) 0.5 and applied to plants at 2 day after planting.

### Plant Material and Growth Conditions

The pea *Pisum sativum* L. *La cry* mutant lines and line segregating *la cry* carrying the mutations in *della1* and *della2* genes were generated as described by Weston et al. (2008) and wild type line is cv. Torsdag. Wild type SGE line and two derived mutants SGEFix^-^-2 and SGEFix^-^-5 [*ipd3*/*cyclops* (*sym33*)] were also used in this study. Gene expression experiments were carried out with seeds sterilized with sulphuric acid for 5 min, washed 3 times with water, transferred on 1% water agar plates and germinated at room temperature in the dark. 4–5 days-old plant seedlings were transferred into pots with vermiculite saturated with Jensen medium (van Brussel et al., 1982), grown in a growth chamber at 21°C at 16 h light/8 h dark cycles, 60% humidity. Fragments of main roots (responsive zone starting from 5 to 6 mm from the root tip) or fragments of main roots with primordia/nodules were collected at 2, 9, and 14 days after inoculation (2, 9, and 14 dai). Fragments of non-inoculated main roots were collected at the same developmental stages. Plant material for gene expression studies was immediately immersed in liquid nitrogen and stored in -80°C freezer.

### RNA Extraction, cDNA Synthesis, and Quantitative PCR

Plant material was ground with a mortar and pestle to a fine powder in liquid nitrogen. Approximately 50–100 mg of ground tissue was used for RNA extraction, as previously described (Dolgikh et al., 2017). 1–2.5 μg of total RNA was used to synthesize cDNA with the RevertAid Reverse Transcriptase (Thermo Fisher Scientific, United States). cDNA samples were diluted to a total volume of 200 μl. For gene expression quantification, the following primer pairs were used (Supplementary Table S1). All primers were acquired from Evrogen company^1^. 2 μl of cDNA was used for quantitative real-time PCR using Bio-Rad iQ Sybr master mix (Bio-Rad Laboratories, United States) following the manufacturer’s recommendations and run on a CFX-96 real-time PCR detection system with C1000 thermal cycler (Bio-Rad Laboratories). All reactions were done in triplicate and averaged. Cycle threshold (Ct) values were obtained using the accompanying software and data were analyzed according to the 2^-ΔΔCt^ method (Livak and Schmittgen, 2001). All primer pairs (Supplementary Table S2) were designed using the Vector NTI program and produced by Evrogen^2^. The gene expression was normalized against the constitutively expressed ubiquitin gene in pea. The gene expression in experiments with wild type and mutants was determined with several biological samples (*n* = 4–8 plants). For temporal *BELL1* gene expression, each replicate contained tissue of 3–4 plants and experiment was repeated three times.

### Cloning of *PsDELLA1, PsKNOX3*, and *PsBELL1* for Yeast Transformation

Full-length *PsDELLA1, PsKNOX3*, and *PsBELL1* coding sequences were obtained by amplification of cDNA cv. Finale or cv. Sparkle using specific PCR primer pairs flanking with attB1 and attB2 sequences or CACC in forward primer (Supplementary Table S2) for subsequent cloning in pDONR221 or pENTRY-TOPO vectors (Thermo Fisher scientific, United States) according to manufacturer’s protocol.

At the next stage they were finally subcloned into the destination vectors pDEST22 (PREY) or pDEST32 (BAIT) vectors using the LR clonase enzyme (Thermo Fisher scientific). All verified constructs were transferred into MaV203 yeast strain (Thermo Fisher scientific).

Full-length *PsNSP2* and *PsIPD3*/*CYCLOPS* coding sequences or partial coding sequences upto stop-codon corresponding to those in RisNod14, E69 *nsp2* (*sym7*) or SGEFix^-^-5 [*ipd3/cyclops* (*sym33*)] mutants were obtained by amplification of cDNA cv. Finale or Sparkle using specific PCR primer pairs flanking with attB1 and attB2 sequences followed by cloning into pDEST22 (PREY) or pDEST32 (BAIT) vectors.

### Yeast Two-Hybrid Assay (GAL4 Transcription Factor-Based Assay)

The *S. cerevisiae* strain MaV203 (Thermo Fisher scientific) was transformed simultaneously with pDEST22 and pDEST32 vectors for GAL4 based selection. To transform the *S. cerevisiae* MaV203, the protocol for preparation of chemically competent cells was used (Thermo Fisher scientific). A few pairs of vectors (pEXP32/Krev1 and pEXP22/RalGDS-wild type, pEXP22/RalGDS-m1 and pEXP22/RalGDS-m2) suggested by the manufacturer as controls were used for strong, weak and not detectable interactions. Analysis of interaction was conducted on selective media like SC –LT (without leucine and tryptophan), SC –LTH + 3AT (without leucine, tryptophan and histidine plus 3-amino-1,2,4-triazole, SC –LTU (without leucine, tryptophan and uracil).

### Statistical Methods and Computer Software

Multiple alignment of nucleotide sequences was performed using Clustal W (Thompson et al., 1994) using Vector NTI Advance 10 (InforMax^3^). MEGA6 was used to generate graphic output of phylogenetic tree (Hall, 2013). One-way ANOVA and Tukey’s test were used to compare gene expression levels.

## Results

### Transcription Levels of *PsKNOX* and *PsBELL* Genes in Pea *La cry-s* (*DELLA1 della2*) and *la cry-s* (*della1 della2*) Mutants

Previously nine *PsKNOX* genes were identified in pea *P. sativum* L. (Hofer et al., 2001; Zhou et al., 2014; Azarakhsh et al., 2015). Their expression pattern was estimated during nodulation and showed stimulation of *PsKNOX3, PsKNOX5, PsKNOX9*, and *PsKNOX10* genes upon nodule development starting from 7 to 9 days after inoculation (7–9 dai) (Azarakhsh et al., 2015).

**FIGURE 1:**
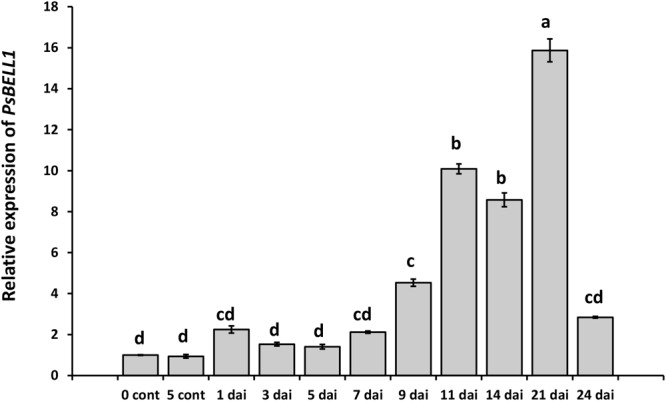
Analysis of *PsBELL1* expression at the various stages of symbiosis development in cv. Torsdag. The roots of non-inoculated plants (NI) have been used as a control. The relative expression was normalized against the constitutively expressed ubiquitin and actin genes. Data are averages ± SEM of three technical repeats. The graphs show the results of one biological experiment, representative for three independent experiments. Values with different letters are significantly different (*P* < 0.05) as analyzed by one-way ANOVA and the Tukey’s test as *post-hoc* analysis.

The screening of Mt4.0v1 database for model legume *M. truncatula* allowed identifying eleven *MtBELL* genes and eleven homologous genes have been found in pea database^4^ (Supplementary Table S1 and Figure S1). Expression atlas showed that at least one gene PsCam048179 (*PsBELL1*, the closest homologue of Medtr8g078480) is significantly induced in the nodules compared to roots. Indeed, in our experiments the enhanced level of *PsBELL1* has been revealed during nodulation in wild type pea plants of cv. Torsdag (Figure 1). In addition, analysis of transcriptome data for model legume *M. truncatula* showed the activation of some *MtKNOX* and *MtBELL* genes in roots in response to Nod factor treatment (van Zeijl et al., 2015; Jardinaud et al., 2016). It suggests that KNOX and BELL transcription factors may be also involved in regulation of early stages of symbiosis development in legumes.

To investigate whether the *Ps*DELLA proteins are involved in regulation of *Ps*KNOX3, *Ps*KNOX5, *Ps*KNOX9, *Ps*KNOX10, and *Ps*BELL1 transcription factors, the transcription levels of corresponding genes have been analyzed in *LA cry-s* (*DELLA1 della2*) and *la cry-s* (*della1 della2*) mutants in response to inoculation using real-time PCR. The *La cry-s* (*DELLA1 della2*) single mutant after inoculation with *R. leguminosarum* bv. *viciae* showed a nodulation phenotype that did not differ from that of the wild-type, however, the *la cry-s* (*della1 della2*) double mutant formed fewer nodules relative to the wild-type but the nodules that formed on mutant plants appeared be functional and to fix similar amount of nitrogen per gram nodule weight (Ferguson et al., 2011; McAdam et al., 2018).

**FIGURE 2:**
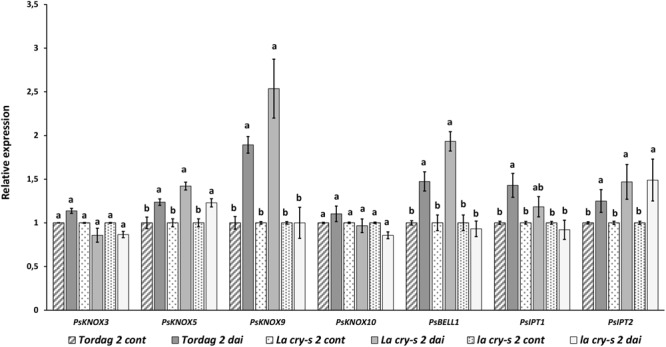
Expression levels of *PsKNOX* and *PsBELL* genes and cytokinin metabolic genes in pea wild type cv. Torsdag and *LA cry-s* (*DELLA1 della2*), *la cry-s* (*della1 della2*) mutants in 2 days after inoculation (2 dai). The expression was normalized against the constitutively expressed ubiquitin gene. For each gene, the transcript level in non-inoculated roots of wild type or mutants was set to 1 (control), and the level in inoculated wild type or mutants was calculated relative to the control values. Data are averages ± SEM (*n* = 5–6 plants of wild type or mutant combined from two independent experiments). Values with different letters are significantly different (*P* < 0.05) as analyzed by one-way ANOVA and the Tukey’s test as *post-hoc* analysis.

At the early stages of symbiosis development the stimulation of two pea *PsKNOX* and *PsBELL* genes, the *PsKNOX9* and *PsBELL1*, was revealed in wild type and *LA cry-s* (*DELLA1 della2*) mutant roots in response to inoculation (2 dai) (Figure 2). In contrast, analysis of mutants showed the *PsKNOX9* and *PsBELL1* expression was not significantly elevated in inoculated *la cry-s* (*della1 della2*) mutant (Figure 2). This suggests that *PsKNOX9* and *PsBELL1* may be involved in the control of early steps of symbiosis in pea and the *Ps*DELLA1 and *Ps*DELLA2 proteins may directly or indirectly influence their activation. Since the effect was more pronounced, when both *della1 della2* genes were impaired, it may indicate that the PsDELLA1 could play a more significant role in symbiosis regulation. At the same time it also could be connected with the functional redundancy of *Ps*DELLA1 and *Ps*DELLA2 proteins in pea.

**FIGURE 3:**
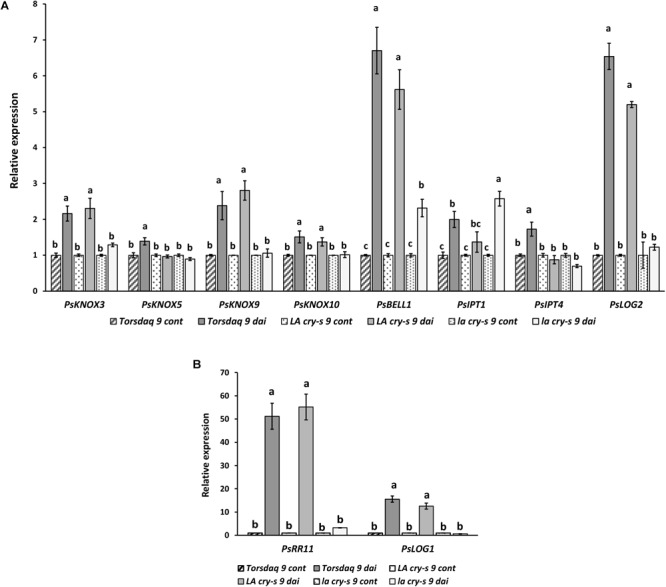
Expression levels of *PsKNOX* and *PsBELL* genes and cytokinin metabolic and response genes in pea wild type cv. Torsdag and *LA cry-s* (*DELLA1 della2*), *la cry-s* (*della1 della2*) mutants in comparison with inoculated wild type in 9 days after inoculation (9 dai). The expression was normalized against the constitutively expressed ubiquitin gene. Panels **A** and **B** represent genes with various level of expression. For each gene, the transcript level in non-inoculated roots of wild type or mutants was set to 1 (control), and the level in inoculated wild type or mutants was calculated relative to the control values. Data are averages ± SEM (*n* = 6–8 plants of wild type or mutant combined from three independent experiments). Values with different letters are significantly different (*P* < 0.05) as analyzed by one-way ANOVA and the Tukey’s test as *post-hoc* analysis.

**FIGURE 4:**
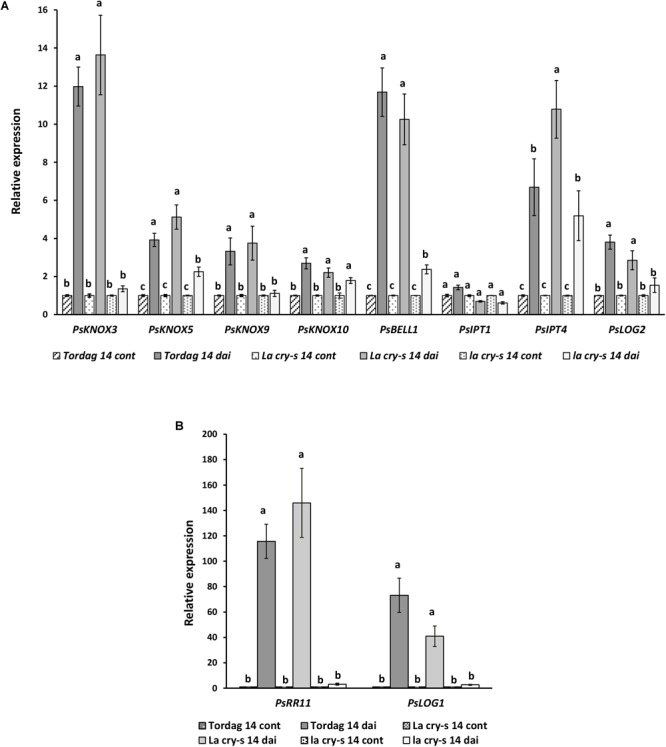
Expression levels of *PsKNOX* and *PsBELL* genes and cytokinin metabolic genes in pea wild type cv. Torsdag and *LA cry-s* (*DELLA1 della2*), *la cry-s* (*della1 della2*) mutants in comparison with inoculated wild type in 14 days after inoculation (14 dai). Panels **A** and **B** represent genes with various level of expression. For each gene, the transcript level in non-inoculated roots of wild type or mutants was set to 1 (control), and the level in inoculated wild type or mutants was calculated relative to the control values. Data are averages ± SEM (*n* = 4–6 plants of wild type or mutant combined from two independent experiments). Values with different letters are significantly different (*P* < 0.05) as analyzed by one-way ANOVA and the Tukey’s test as *post-hoc* analysis.

To verify the impact of DELLA proteins on later stages of nodulation, which includes infection thread formation and growth, nodule primordia initiation and development, we have performed the analysis with wild type and mutants in 9 and 14 dai (Figure 3, 4). As previously reported (Azarakhsh et al., 2015), it was also found that *PsKNOX3, PsKNOX5, PsKNOX9*, and *PsKNOX10* expression was upregulated in wild type pea plants in the process of nodulation. In contrast to wild type, there was only little increase in *PsKNOX3* and *PsKNOX9* expression in *la cry-s* (*della1 della2*) mutant in 9 and 14 dai, while the *PsKNOX5* showed a moderate up-regulation in double *la cry-s* (*della1 della2*) mutant in 14 dai (Figure 3, 4). At the same time, the up-regulation of *PsKNOX10* expression seen in wild type plants 14 dai was also observed in *la cry-s* (*della1 della2*) mutants (Figure 4). Therefore, our study identified *PsKNOX3* and *PsKNOX9* transcription as potentially regulated by *Ps*DELLA1 and *Ps*DELLA2 proteins in pea *P. sativum* L. during nodulation. The *Ps*DELLA1 and *Ps*DELLA2 proteins may also be involved in an additional stimulation of the *PsKNOX5* expression in pea plants. Similarly, the significant upregulation in the expression of *PsBELL1* seen 9 and 14 dai in wild type was not significantly elevated in *la cry-s* (*della1 della2*) mutant in response to inoculation (Figure 3, 4). It suggests that *Ps*DELLA1 and *Ps*DELLA2 proteins may also promote the expression of *PsBELL1* gene in pea.

### Regulation of *PsLOG* and *PsIPT* Genes in Pea *LA cry* and *la cry* Mutants

It was shown that expression of some *PsLOG* and *PsIPT* genes may depend on the *Ps*KNOX3 transcription factor in pea (Azarakhsh et al., 2015). To investigate a possible link between regulation of transcription factors by DELLA and possible targets of KNOX transcription factors, we have examined the *PsLOG1, PsLOG2, PsIPT1, PsIPT2, PsIPT3*, and *PsIPT4* (all of them encode enzymes that belong to adenosine phosphate-IPTs) expression in *LA cry* and *la cry* mutants during nodulation (2, 9, and 14 dai) (Figure 3, 4), because their up-regulation was shown upon nodulation in pea (Azarakhsh et al., 2015; Dolgikh et al., 2017). However, as the level of *IPT3* was very low in cv. Torsdag and both mutants it was not included in the analysis. In addition, we have also analyzed the transcription levels of the cytokinin-responsive *PsRR11* gene, which is most significantly activated in pea root in response to inoculation in comparison with other *PsRRs* like *PsRR4, PsRR5, PsRR8*, and *PsRR9* (Dolgikh et al., 2017).

Early stages of symbiosis development may be connected with stimulation of *PsIPT1* (the closest homolog of Medtr4g117330) and *PsIPT2* (the closest homolog of Medtr2g022140) genes in pea (Dolgikh et al., 2017). However, we did not find essential differences in expression of these genes between wild type cv. Torsdag and *LA cry* and *la cry* mutants in 2 dai (Figure 2). As it was shown previously, up-regulation of the *PsLOG1, PsLOG2* and *PsIPT1, PsIPT3* and *PsIPT4* genes involved in CK biosynthesis may take place at later stages of symbiosis development (Azarakhsh et al., 2015; Dolgikh et al., 2017). Our experiments showed that in contrast to stimulation in cv. Tosdag (wild type), the upregulation in expression of the *PsLOG1, PsLOG2* genes and cytokinin-responsive *PsRR11* genes observed in wild type plants was not observed in the double mutant *la cry* at 9 and 14 dai, suggesting that *Ps*DELLA1 and *Ps*DELLA2 proteins might be required for this upregulation. The pattern expression of *PsIPT1* and *PsIPT4* (the closest homolog of Medtr1g110590) expression in response to inoculation was similar in wild type, *LA cry* and *la cry* mutants in 9 and 14 dai (Figure 4), suggesting *Ps*DELLA is not essential for the regulation of these genes. A lack of correlation between the change in expression of *PsKNOX3* gene and the *PsIPT4* gene was previously shown in our experiments (Azarakhsh et al., 2015).

### DELLA Proteins Are Able to Regulate GA Metabolic Genes

**FIGURE 5:**
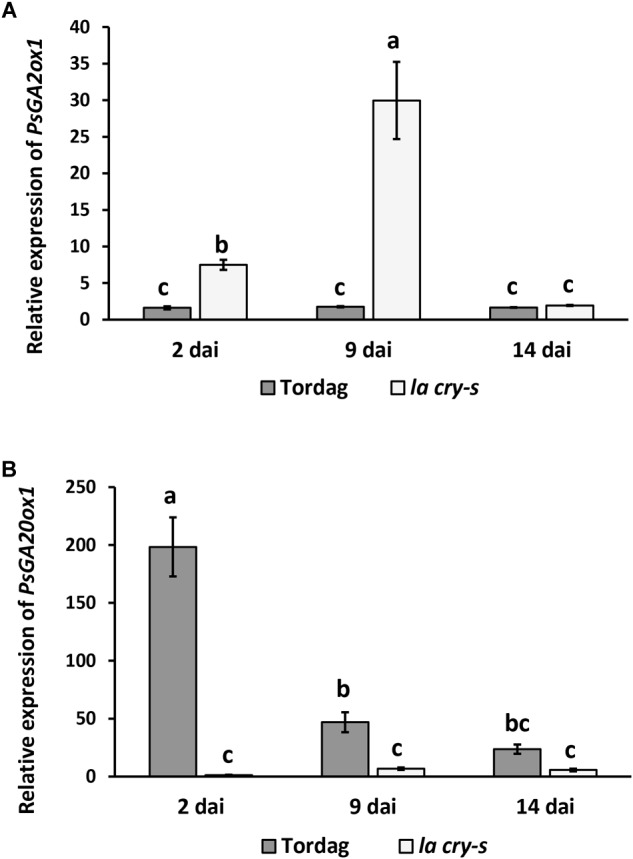
*PsGA2ox1* **(A)** and *PsGA20ox1* **(B)** gene expression in roots of wild-type cv. Torsdag and *la cry-s* (*della1 della 2*) mutant plants inoculated by *R. leguminosarum bv. viciae* 3841 in 14 dai. For each gene, the transcript level in non-inoculated roots of wild type or mutants was set to 1 (control), and the level in inoculated wild type or mutants was calculated relative to the control values. Finally, the expression in wild type was presented relative to the level in mutant. Data are averages ± SEM (*n* = 4–8 plants of wild type or mutant combined from two-three independent experiments). Values with different letters are significantly different (*P* < 0.05) as analyzed by one-way ANOVA and the Tukey’s test as *post-hoc* analysis.

It was previously suggested that *Ps*DELLA1 and *Ps*DELLA2 proteins may regulate GA metabolism modulating the expression of corresponding genes in pea (Weston et al., 2008). Indeed, a significant reduction of expression of GA biosynthesis (*PsGA20ox1*) gene, but stimulation of GA deactivation (*PsGA2ox1*) gene were found in non-inoculated roots of 6 days-old seedlings of *la cry-s* mutant compared with wild type (Weston et al., 2008). To estimate the expression dynamics of GA metabolic genes during nodulation, the transcription levels of the *PsGA20ox1* and *PsGA2ox1* genes have been measured in wild type and *la cry-s* mutant in 2, 9, and 14 dai using real-time PCR (Figure 5). We found a strong reduction in expression of the GA biosynthesis gene *PsGA20ox1* at all stages in *la cry-s* mutant compared to wild type, but the most significant differences were found at early stages (2 and 9 dai). At the same time in *la cry-s* a strong stimulation of the GA deactivation gene *PsGA2ox1* was found starting from 2 dai and reaching the highest level at 9 dai. This suggests that significant changes in GA metabolism may take place at initial stages of symbiosis development. It could not be excluded that the DELLA proteins are involved in this regulation, since previous studies have shown an important role of DELLA proteins in establishment of GA homeostasis (Zentella et al., 2007).

### Analysis of *Ps*DELLA1 Protein Interaction With *Ps*KNOX3, *Ps*BELL1 in Yeast Two-Hybrid System (Y2H)

To determine a possible interaction between pea DELLA1 protein and the KNOX and BELL transcription factors, we performed a yeast two-hybrid (Y2H) assay with the fusion proteins. Since the most significant role may play *Ps*KNOX3 and *Ps*BELL1 transcription factors, the analysis was performed with *Ps*DELLA1-GAL4-DNA-binding domain (*Ps*DELLA1-GAL4-DBD) and *Ps*KNOX3-GAL4 activation domain (*Ps*KNOX3-GAL4-AD) as well as *Ps*BELL1-GAL4 activation domain (*Ps*BELL1-GAL4-AD).

**FIGURE 6:**
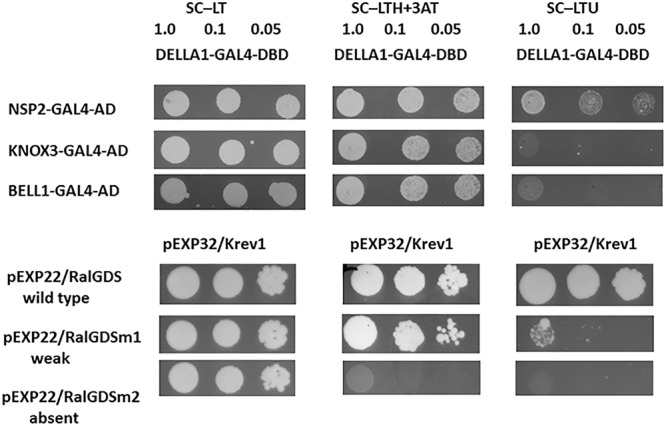
Yeast two-hybrid assay (GAL4 transcription factor-based) for DELLA1 protein and KNOX3 and BELL1 transcription factors. The *S. cerevisiae* strain MaV203 was transformed simultaneously with pDEST22 and pDEST32 vectors for GAL4 activation domain (AD) or GAL4 DNA binding domain (DBD). The pairs of vectors pEXP32/Krev1 and pEXP22/RalGDS-wild type, pEXP22/RalGDS-m1 and pEXP22/RalGDS-m2 were used for strong, weak and not detectable interactions. Yeast growth was tested on SC medium without leucine, tryptophan and histidine with 3-amino-triazole (SC –LTH + 50 mM 3 AT) and SC without leucine, tryptophan and uracil (SC – LTU).

**FIGURE 7:**
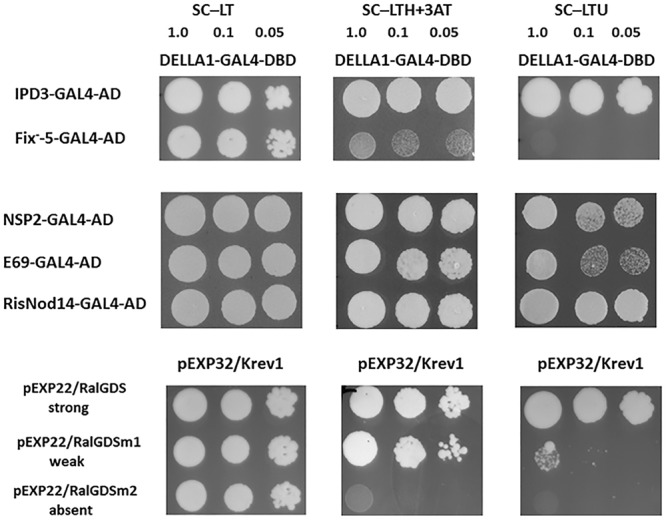
Yeast two-hybrid assay (GAL4 transcription factor-based) for DELLA1 protein and NSP2 and IPD3/CYCLOPS transcription factors and their truncated variants corresponding to those in E69 (*nsp2*/*sym7-1*), RisNOD14 (*nsp2*/*sym7-2*) and SGEFix^-^–5 (*ipd3/cyclops/sym33-2*). AD – activation domain of GAL4, DBD – DNA binding domain of GAL4. The pairs of vectors pEXP32/Krev1 and pEXP22/RalGDS-wild type, pEXP22/RalGDS-m1 and pEXP22/RalGDS-m2 were used for strong, weak and not detectable interactions. Yeast growth was tested on SC medium –LTH + 50 mM 3 AT and SC – LTU.

Previously the capacity of DELLA proteins to promote CCaMK–IPD3/CYCLOPS and NSP2–NSP1 complexes formation was shown for model legumes *Lotus japonicus* and *M. truncatula* (Fonouni-Farde et al., 2016; Jin et al., 2016). To verify our results, we have also performed the analysis for *Ps*DELLA1-GAL4-DNA-binding domain (*Ps*DELLA1-GAL4-DBD) and *Ps*NSP2-GAL4 activation domain (*Ps*NSP2-GAL4-AD), *Ps*IPD3/CYCLOPS-GAL4 activation domain (*Ps*IPD3/CYCLOPS-GAL4-AD). Indeed, the analysis of yeast growth on selection media showed that *Ps*DELLA1 was able to form complex with *Ps*NSP2 (SYM7) as well as *Ps*IPD3/CYCLOPS (SYM33) in Y2H (Figure 6, 7). These results were in accordance with results for *L. japonicus* and *M. truncatula*.

At the same time we did not reveal a strong interaction between *Ps*DELLA1 and *Ps*KNOX3, *Ps*DELLA1 and *Ps*BELL1 in this system (Figure 6). This conclusion can be made on the basis of the growth of MaV203 yeast cells producing both *Ps*DELLA1-GAL4-DBD and *Ps*KNOX3-GAL4-AD, as well as *Ps*DELLA1-GAL4-DBD and *Ps*BELL1-GAL4-AD on SC –LTH + 50 mM 3 AT, but not on SC – LTU. Therefore, we have not received the evidences of a strong direct interaction between *Ps*DELLA1 and *Ps*KNOX3 or *Ps*BELL1 proteins.

### The Interplay Between DELLA, KNOX3, and IPD3/CYCLOPS Regulators

Since DELLA appeared to be important in the regulation of *PsKNOX3* and *PsBELL1* expression in our experiments, we suggested that other transcription factors may be involved in transactivation of *PsKNOX3* and *PsBELL1* in pea. Among of them, the *Ps*NSP2 and *Ps*IPD3/CYCLOPS transcription factors are the most probable candidates for indirect activation of *PsKNOX* and *PsBELL* genes. Moreover, the interaction between *Ps*DELLA1 and *Ps*NSP2, *Ps*IPD3/CYCLOPS was shown in our experiments using Y2H system.

Previously a similar effect was found for *Mt*ERN1 transcription factor, which is activated by *Mt*DELLA1 through *Mt*NSP2/*Mt*NSP1 complex formation and binding with promoter of *MtERN1* (Fonouni-Farde et al., 2016). The binding with promoter induced the expression of *MtERN1* and its target the *MtENOD11* gene in *M. truncatula*.

Several pea mutants impaired in *nsp2* (*sym7*) and *ipd3/cyclops* (*sym33*) genes are available. The pea mutants E69 and RisNod14 are impaired in *nsp2* (*sym7*) gene and demonstrates the Nod^-^ phenotype (Engvild, 1987; Kneen et al., 1994; Walker and Downie, 2000; Tsyganov et al., 2002; Kalo et al., 2005). The pea mutants SGEFix^-^-2 and SGEFix^-^-5 are impaired in *ipd3/cyclops* (*sym33*) gene (Fix^-^ phenotype) and able to form rare nodules which remained uninfected (Tsyganov et al., 1998; Voroshilova et al., 2009).

To check the interaction between *Ps*DELLA1 protein and truncated *Ps*NSP2 proteins corresponding to those in E69 and RisNod14 mutants as well as truncated *Ps*IPD3/CYCLOPS protein corresponding to that in SGEFix^-^-5 we performed analysis using Y2H system. This revealed that both truncated *Ps*NSP2 proteins were still able to interact with *Ps*DELLA1, but truncated *Ps*IPD3/CYCLOPS failed to interact with *Ps*DELLA1 (Figure 7). This demonstrates that interruption of mutual recognition between *Ps*DELLA1 and *Ps*IPD3/CYCLOPS may result in signal transduction failure in *ipd3/cyclops* pea mutant. In case of two *nsp2* (*sym7*) mutants the recognition between *Ps*DELLA1 and *Ps*NSP2 still takes place, but seems like the both truncated *Ps*NSP2 are not able to interact with *Ps*NSP1 or other transcription factors and subsequent signal transduction is blocked.

**FIGURE 8:**
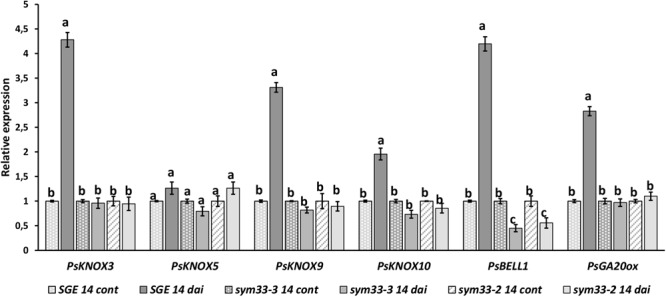
Expression analysis of *PsGA20ox, PsKNOX*, and *PsBELL* genes in wild type cv. SGE and SGEFix^-^–2 (*sym33–3*), SGEFix^-^–5 (*sym33–2*) mutants 2 weeks after inoculation. Transcript levels of genes were normalized against ubiquitin gene. For each gene, the transcript level in non-inoculated roots of wild type or mutants was set to 1 (control), and the level in nodules of wild type or mutants was calculated relative to the control values. Data are averages ± SEM (*n* = 6–8 plants of wild type or mutant combined from two independent experiments). Values with different letters are significantly different (*P* < 0.05) as analyzed by one-way ANOVA and the Tukey’s test as *post-hoc* analysis.

Similarly, with *la cry-s* (*della1 della2*) mutant, a reduced number of nodules was found in *ipd3/cyclops* (*sym33*) mutants, although the nodules of *ipd3/cyclops* (*sym33*) were not effective (Tsyganov et al., 1998; Ovchinnikova et al., 2011). Taking this into account, we might expect that in *ipd3/cyclops* (*sym33*) mutants the expression of *PsKNOX3* and *PsKNOX9* genes may be misregulated during infection as observed in *la cry-s* (*della1 della2*) mutant. To check this, we analyzed the expression of *PsKNOX3, PsKNOX5, PsKNOX9, PsKNOX10*, and *PsBELL1* in roots bearing nodules (14 dai) from pea mutants SGEFix^-^-5 and SGEFix^-^-2 [*ipd3/cyclops* (*sym33*)]. Indeed, the SGEFix^-^-5 and SGEFix^-^-2 [*ipd3/cyclops* (*sym33*)] did not upregulated the expression of these genes as significantly as found in wild type plants (Figure 8). Our results suggest that the induction of *PsKNOX3, PsKNOX9*, and *PsBELL1* expression may require DELLA-dependent activation and analysis of *ipd3/cyclops* (*sym33*) mutants suggests this may be via interaction with *Ps*IPD3/CYCLOPS transcription factor. In addition, a reduced transcription level of the GA biosynthesis gene *PsGA20ox1* was found in *ipd3/cyclops* (*sym33*) mutants compared to wild type in 14 dai using real-time PCR. Decreased level of *PsGA20ox1* expression in *della1 della2* and *ipd3/cyclops* (*sym33*) mutants may reflect the importance of GA for nodule development in pea. Upregulation of GA metabolic genes may be also connected with activation of *Ps*KNOX and *Ps*BELL transcription factors.

## Discussion

Multiple studies have shown an important role of CKs in regulation of infection process and nodule organogenesis during legume-rhizobial symbiosis. In addition to CKs, the GAs also have a strong impact on symbiosis development. Indeed, transcriptome analyses revealed the substantial alterations in the expression of genes encoding enzymes of CK and GA biosynthesis and degradation during symbiosis initiation and development in legume plants (Breakspear et al., 2014; van Zeijl et al., 2015; Jardinaud et al., 2016). The antagonistic relationships between CKs and GAs were shown in many developmental processes and were also described in nodulation in *M. truncatula* and *L. japonicus* (Maekawa et al., 2009; Jin et al., 2016). It was shown that the number of spontaneous nodules induced by overexpression of *LjLHK1*/*MtCRE1* genes encoding the receptor to CKs was decreased upon the addition of GAs (Maekawa et al., 2009; Jin et al., 2016). This effect was depended on DELLA proteins, suggesting that DELLAs were required for spontaneous nodule formation induced by CKs, but GAs inhibited this process.

Recent study has also revealed that GA pretreatment reduced the Nod factor-induced CK primary response in the epidermis and in the outer cortex in *M. truncatula* as well as CK response gene expression, the *MtRRA8, MtRRA9, MtRRA11* (Fonouni-Farde et al., 2017). It has been also shown that pretreatment with GAs may decrease the amount of some pools of bioactive CKs in roots in *M. truncatula* (Fonouni-Farde et al., 2017).

Indeed, exogenous GAs has a negative effect on the number of nodules in *M. truncatula* and *L. japonicus* and this was also seen at high concentrations of GA in pea (Ferguson, 2005; Maekawa et al., 2009; Fonouni-Farde et al., 2016; Jin et al., 2016). However, at lower concetrations GA actually enhanced nodule number in pea and Sesbania (Ferguson, 2005; Lievens et al., 2005). This indicates a more complicated role for GAs during nodulation. While this apparent paradox may be explained by stating that there is an optimal level of GAs for nodulation overall, this may in fact be due to GAs exerting different effects on specific stages of nodulation, inhibiting infection but promoting nodule organogenesis (Ferguson, 2005; Ferguson et al., 2011; McAdam et al., 2018). This is similar to the dual role proposed for CKs during nodulation (Gamas et al., 2017).

This regulation could be achieved by interaction between DELLA proteins and plant regulators controlling the content of CKs and GAs in plants. Since the participation of KNOX and BELL transcription factors in regulation of CK and GA metabolic genes is well-known for plants, the pea *la cry-s* (*della1 della2*) mutants have been used to examine the effect of these proteins on the expression of *PsKNOX* and *PsBELL* genes during nodulation. Indeed, we showed failure to upregulate the expression of *PsKNOX9* and *PsBELL1* genes at early stages of symbiosis development in inoculated roots of pea *la cry-s* (*della1 della2*) mutant. Although we did not find a definite correlation between *Ps*DELLAs function and regulation of some *PsIPT* genes at early stages, but influence on other CK metabolic genes could not be excluded. Recently the involvement of GAs in stimulation of CKs degradation via cytokinin oxidase *Mt*CKX3 has been revealed in *M. truncatula* (Fonouni-Farde et al., 2017). An alternative explanation might be that *PsKNOX9* and *PsBELL1* influence GA metabolic genes at early stages of symbiosis, as revealed significant changes in GA metabolism may take place at initial stages of symbiosis development and *Ps*DELLA proteins are involved in this regulation.

At later stages of symbiosis development the up regulation of expression of *PsKNOX3, PsKNOX9*, and *PsBELL1* was shown to be attenuated in pea *della1 della2* mutant. However, up regulation in the expression of other *PsKNOX* genes, the *PsKNOX5* and *PsKNOX10*, was only slightly reduced or similar in *della* mutant compared to wild type. Consistently, pea *la cry-s* (*della1 della2*) mutant showed reduced upregulation in the expression of the CK synthesis (*PsLOG1, PsLOG2*) and signaling (*PsRR11*) genes during nodulation compared to wild type. One interpretation is that *Ps*DELLA proteins can regulate the CK metabolism via *Ps*KNOX and *Ps*BELL transcription factors during nodule development. Upregulation of CK metabolic genes has previously been shown to be due to activation of KNOX and BELL transcription factors (Yanai et al., 2005; Azarakhsh et al., 2015).

As has been shown in analysis of model legumes, the DELLA proteins may regulate a number of transcription factors during nodulation. In some cases the DELLA proteins interact with individual transcription factors including NSP2, IPD3/CYCLOPS and NF-YA1 (direct activation), in other cases their effect may be indirect. Since DELLAs don’t have a DNA-binding motif, they may induce binding of activated transcription factors with promoter of genes encoding other transcription factors like ERN1 (Fonouni-Farde et al., 2016).

To verify the effect of *Ps*DELLA proteins on *Ps*KNOX3 and *Ps*BELL1 transcription factors, the possibility of their interaction has been investigated using Y2H system. However, our studies did not reveal a strong direct interaction between *Ps*DELLA1 and *Ps*KNOX3 or *Ps*BELL1 in this heterologous system. We suggested that *Ps*DELLA1 may promote the expression *PsKNOX3* and *PsBELL1*, probably through other known transcription factors. This function may be related to IPD3/CYCLOPS and NSP2 transcription factors, because DELLA proteins can activate them during nodulation (Floss et al., 2016; Fonouni-Farde et al., 2016; Jin et al., 2016). We tested the interaction between *Ps*DELLA1 and truncated proteins corresponding to those in pea *ipd3/cyclops* (*sym33*) and *nsp2* (*sym7*) mutants. Surprisingly, only *Ps*IPD3/CYCLOPS truncated protein failed to interact with *Ps*DELLA1, but *Ps*DELLA1 still interacted with both *Ps*NSP2 truncated proteins. It suggests that in both *ipd3/cyclops* (*sym33*) mutants the interaction between *Ps*DELLA1 and *Ps*IPD3/CYCLOPS may be critical for subsequent signal transduction. Since similar trends for reduced ability to up regulate the expression of *PsKNOX3* and *PsKNOX9* transcription levels were found in the nodules of *ipd3/cyclops* (*sym33*) mutants as seen in *della* mutants compared with wild type, the *Ps*DELLA proteins may promote the expression of *PsKNOX3* and *PsBELL1* genes via the *Ps*IPD3/CYCLOPS transcription factor. Additional experiments that could explore this further include analyzing the binding capacity of *Ps*IPD3/CYCLOPS with promoters of the *PsKNOX3* and *PsKNOX9* genes in pea plants and the subsequent effect on target genes.

As previously reported (Ferguson et al., 2011), *la cry-s* mutant had significantly fewer nodules than wild-type plants. Similarly, *ipd3/cyclops* (*sym33*) mutants had a considerably reduced number of nodules (Tsyganov et al., 1998; Ovchinnikova et al., 2011). Therefore, it may suggest the involvement of *Ps*DELLA proteins and *Ps*IPD3/CYCLOPS transcription factor in initiation of nodule organogenesis and regulation of nodule number in pea plants. As it was shown for model legume plants *M. truncatula* and *L. japonicus*, the DELLA proteins are involved in activation of IPD3/CYCLOPS transcription factor. Our findings revealed that DELLA proteins and IPD3/CYCLOPS also influence the expression of pea *KNOX* and *BELL* genes encoding transcription factors.

The infection thread formation was significantly reduced in DELLA-deficient pea *la cry-s* double mutants compared with wild-type plants (McAdam et al., 2018). Similar to *la cry-s*, pea *ipd3/cyclops* (*sym33*) also showed impaired invasion of the nodule primordia at early stages (Voroshilova et al., 2009). The most probable explanation that impaired infection may influence the signal exchange between epidermis and cortical cells resulting in decreased number of nodule primordia in both type of mutants. Indeed, the experiments in which the expression of GA-insensitive *Mt*DELLA1 was restricted by epidermis showed that it was able to trigger the spontaneous nodule formation in spatially distant cortical cells in absence of rhizobia (Fonouni-Farde et al., 2017). It suggests that some diffusive factors may be involved in signal transduction in this case. Since the KNOX and BELL transcription factors may act in non-cell autonomous manner, additional experiments should explore a possible role of KNOX and BELL as such diffusive factors.

## Author Contributions

AD and ED conceived the study and designed the experiments. AD and AK performed the experiments and analyzed the data. IT supervised. AD, EF, and ED wrote the manuscript.

## Conflict of Interest Statement

The authors declare that the research was conducted in the absence of any commercial or financial relationships that could be construed as a potential conflict of interest.

## Footnotes

1 www.evrogen.com

2 http://www.evrogen.com

3 http://www.informaxinc.com

4 http://bios.dijon.inra.fr

## Abbreviations

*IPT*: isopentenyl transferase
*LOG*: lonely guy
qRT-PCR: quantitative reverse transcription PCR
*RR*: response regulator
Y2H: yeast two hybrid system
