# Supplementary material for: The DELLA Proteins Influence the Expression of Cytokinin Biosynthesis and Response Genes During Nodulation
Source: Front Plant Sci. 2019 Apr 9;10:432. doi: 10.3389/fpls.2019.00432 (PMC6465611; doi:10.3389/fpls.2019.00432)
Supplement: Supplementary file 1 [file Data_Sheet_1.pdf]

frontiers

Supplementary Material

The influence of DELLA proteins on gibberellins and cytokinins during nodulation

Dolgikh A.V.[1], Kirienko A.N.[1], Tikhonovich I.A., Foo E.[2] and Dolgikh E.A.[1,*]

*** Correspondence:** Corresponding Author: Dolgikh E. A., e-mail: dol2helen@yahoo.com

Table S1. The identification numbers of *M. truncatula* and *P. sativum BELL* genes

Medicago truncatula<br>Mt4.0v1	*Pisum sativum*<br>cv. Cameor<br>http://bios.dijon.in ra.fr/
Medtr5g018860	PsCam035874
Medtr1g023050	PsCam048223
Medtr1g057790	PsCam045115
Medtr3g112290	PsCam044261
Medtr3g112300	PsCam026823
Medtr4g019450	PsCam045494
Medtr4g051532	PsCam034469
Medtr7g065050	PsCam036616
Medtr7g106320	PsCam049248
Medtr8g078480 (*BELL1*)	PsCam048179
Medtr8g098815	PsCam056084

Figure S1. Phylogenetic tree of the *MtBELL* and *PsBELL* gene families.

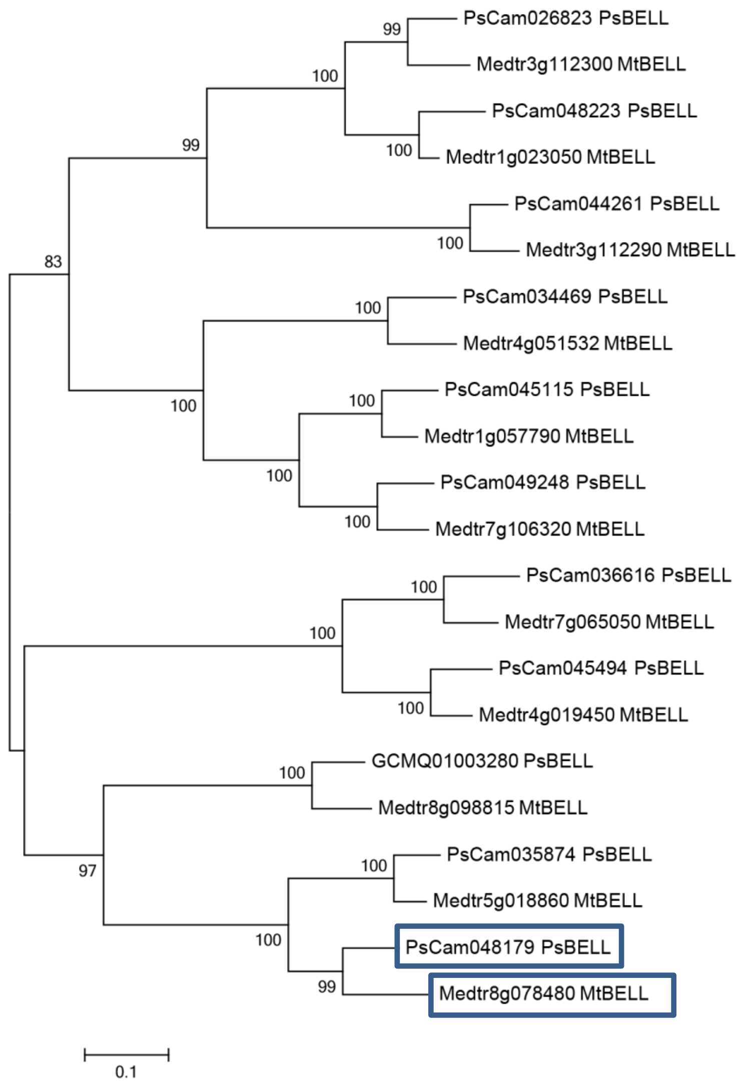

The evolutionary history was inferred by using the Maximum Likelihood method based on the Tamura-Nei model. The branches show the bootstrap values. The *M. truncatula MtBELL1* and *P. sativum* L. *PsBELL1* were presented in the boxes. Evolutionary analyses were conducted in MEGA6. The *BELL*s are named with the first letters of the genus and species: *Ps, Pisum sativum*; *Mt, Medicago truncatula*.

Table S2. List of primers used for PCR.

Gene name	Forward primer	Reverse primer
Ubiquitin	5'-ATGCAGATC/TTTTGTGAAGAC-3'	5'-ACCACCACGG/AAGACGGAG-3'
KNOX3	5'- ATTCCGATCCCAACCAACTCC-3'	5'-ACTTACTCCTCCGTCGCCTTTCAA-3'
KNOX5	5'- ACCTCCGAACCTCACCCATCCT-3'	5'-TGCGCCATTATTTCCCCTTTGTAT-3'
KNOX9	5'-CACTTGGAACCGGAGAGGAGAG-3'	5'-GAGTTGCGATTCGGAGACACG-3'
KNOX10	5'-ACATGCAAAGGCTACTACCGTTCA-3'	5'-AATTCCTCTTCCGTCGTCGTTTTG-3'
BELL1	5'-CTCACGGCGCCTCTCCTG-3'	5'-TGAAATATGCTGCTGCTGCTACTG-3'
IPT1	5'-CAAACAAGGTCACCGAGGAAGAGT-3'	5'-GTGCGGATAAAGACGAATGAAGC-3'
IPT2	5'-CAGTGGCAGCAACATCATCCTC-3'	5'-GCCCCCATTATCACTACCACCTTA-3'
IPT3	5'-AATCACGGGCCACGGACATC-3'	5'-CATCGACCCAAAGACAACAGAAG-3'
IPT4	5'-ATCCCCTTTCATCAACGCAACAA-3'	5'-GTCCACCATTCCCGAGTCAAACAT-3'
LOG1	5'-TGACAAGCCGGTGGGATTAGTAG-3'	5'-ATTATGTGACGAGCATTTGGACTG-3'
LOG2	5'-ACAAGCTGTTCATGATGGTGGTAGA-3'	5'-AGCTTTGTCAATGAATGACAGTAAC-3'
RR11	5'-GGCTTCAGAGACCCAGTTTCAT-3'	5'-TTCAGCTCCCTCTTCCATACAC-3'
